# 2726. A Retrospective Single Center Study to Assess the Risk Factors for Infection with Multi-Drug-Resistant Organisms in Liver Transplant Recipients

**DOI:** 10.1093/ofid/ofad500.2337

**Published:** 2023-11-27

**Authors:** Neeraja Swaminathan, Margaret E McCort, Shalom Frager, Milan Kinkhabwala, Rachel Bartash, Aastha Vasa

**Affiliations:** University of Utah, Salt Lake City, Utah; Montefiore Medical Center / Albert Einstein College of Medicine, Bronx, New York; Montefiore, NY, New York; Montefiore, NY, New York; Montefiore Medical Center, Bronx, NY; Albert Einstein College of Medicine, Bronx NY, Paramus, New Jersey

## Abstract

**Background:**

Infections with multidrug resistant organisms (MDROs) are common in liver transplant recipients. We aimed to understand the prevalence and risk factors for MDRO infections during the peri-transplant period.

**Methods:**

We conducted a retrospective chart review of adults ( >18 years) who underwent deceased donor liver transplant (LT) from Jan 2018-Dec 2020. Demographics and clinical information, including antibiotic use, microbiological data, and adequacy of perioperative antibiotics based on CLSI breakpoints, were reviewed. Peri-transplant period was defined as 12 months pre- and 3 months post-transplant. MDRO was defined as resistance to one or more classes of antibiotics. Statistical analysis was performed with IBM SPSS 29.0, using Chi2 or Fischer’s exact tests as appropriate for categorical variables and independent t-test for continuous variables.

**Results:**

Baseline demographics for the 121 LT recipients are summarized in Table 1. In the 3 months post-transplant, 47 patients (38.8%) were noted to have positive cultures, of which 22 were MDRO. MDRO distribution for pre- and post-transplant infections is shown in Figure 1. Univariate analyses of predictors for post-transplant MDRO infection are shown in Table 2. Mean length of stay for patients with post-transplant MDRO infection was 65.3 days versus 28.6 days in those without MDRO infection (p< 0.001). Among 36 patients with positive cultures < 1month post-transplant, 55% (n=20) received perioperative antibiotics covering the identified organism. Eleven of 23 patients (47.8%) who received ampicillin-sulbactam (AS) and 3 of 8 patients (37.5%) who received piperacillin-tazobactam (PT) perioperatively developed infections not covered by their respective regimens < 1-month post-transplant. In the first month post-transplant, 17 patients had organisms cultured which were resistant to AS, of which all but one was also resistant to PT.

**Table 1:**
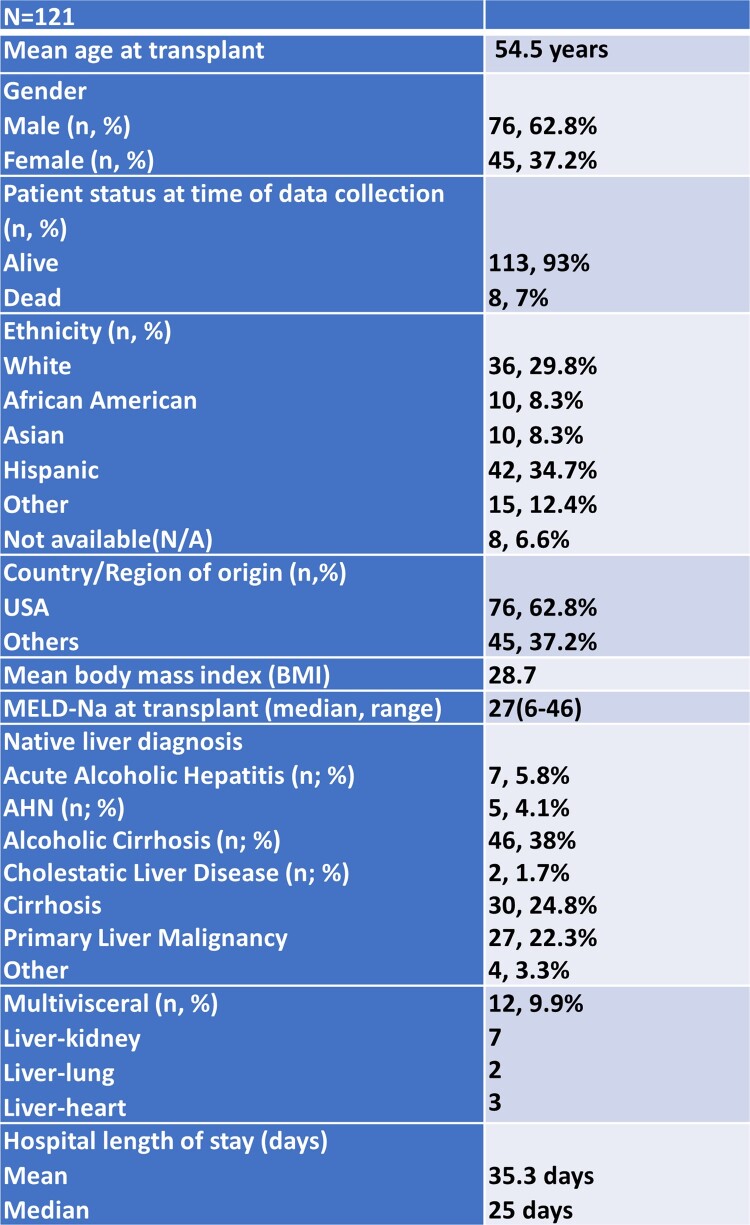
Baseline Demographics

**Figure:**
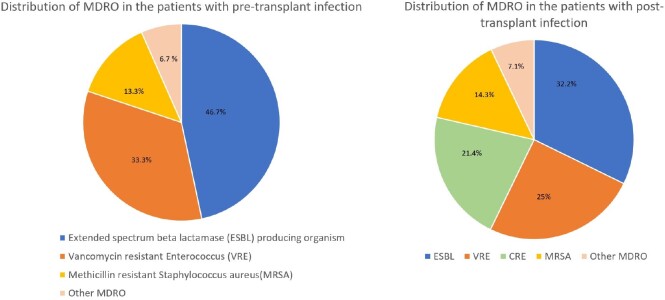

**Figure:**
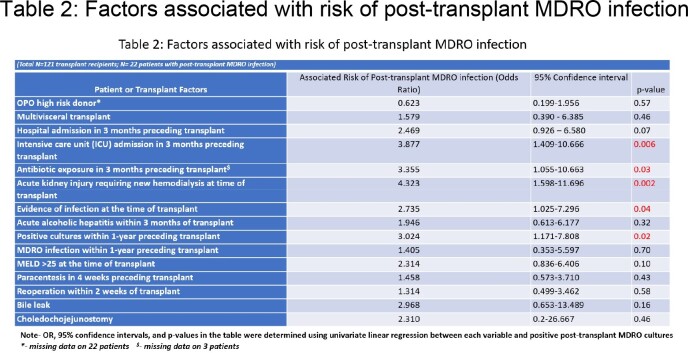

**Conclusion:**

Admission to the intensive care unit, antibiotic use prior to transplant, and longer transplant hospitalizations were associated with MDRO infections. Tailoring of surgical prophylaxis may be indicated for high-risk patients as PT did not offer superior coverage over AS.

**Disclosures:**

**All Authors**: No reported disclosures

